# Brain Ischemic Tolerance Triggered by Preconditioning Involves Modulation of Tumor Necrosis Factor-α-Stimulated Gene 6 (TSG-6) in Mice Subjected to Transient Middle Cerebral Artery Occlusion

**DOI:** 10.3390/cimb46090595

**Published:** 2024-09-10

**Authors:** Chiara Di Santo, Antonio Siniscalchi, Daniele La Russa, Paolo Tonin, Giacinto Bagetta, Diana Amantea

**Affiliations:** 1 Section of Preclinical and Translational Pharmacology, Department of Pharmacy, Health and Nutritional Sciences, University of Calabria, 87036 Rende, Italy; chiara.disanto@unical.it (C.D.S.);; 2 Department of Neurology and Stroke Unit, Annunziata Hospital, 87100 Cosenza, Italy; 3 Regional Center for Serious Brain Injuries, S. Anna Institute, 88900 Crotone, Italy

**Keywords:** cerebral ischemia, neuroprotection, preconditioning, stroke, TSG-6

## Abstract

Ischemic preconditioning (PC) induced by a sub-lethal cerebral insult triggers brain tolerance against a subsequent severe injury through diverse mechanisms, including the modulation of the immune system. Tumor necrosis factor (TNF)-α-stimulated gene 6 (TSG-6), a hyaluronate (HA)-binding protein, has recently been involved in the regulation of the neuroimmune response following ischemic stroke. Thus, we aimed at assessing whether the neuroprotective effects of ischemic PC involve the modulation of TSG-6 in a murine model of transient middle cerebral artery occlusion (MCAo). The expression of TSG-6 was significantly elevated in the ischemic cortex of mice subjected to 1 h MCAo followed by 24 h reperfusion, while this effect was further potentiated (*p* < 0.05 vs. MCAo) by pre-exposure to ischemic PC (i.e., 15 min MCAo) 72 h before. By immunofluorescence analysis, we detected TSG-6 expression mainly in astrocytes and myeloid cells populating the lesioned cerebral cortex, with a more intense signal in tissue from mice pre-exposed to ischemic PC. By contrast, levels of TSG-6 were reduced after 24 h of reperfusion in plasma (*p* < 0.05 vs. SHAM), but were dramatically elevated when severe ischemia (1 h MCAo) was preceded by ischemic PC (*p* < 0.001 vs. MCAo) that also resulted in significant neuroprotection. In conclusion, our data demonstrate that neuroprotection exerted by ischemic PC is associated with the elevation of TSG-6 protein levels both in the brain and in plasma, further underscoring the beneficial effects of this endogenous modulator of the immune system.

## 1. Introduction

When considered separately from other cardiovascular diseases, stroke ranks fifth among all causes of death, behind heart disease, cancer, COVID-19, and unintentional injuries/accidents; moreover, it is the leading cause of disability-adjusted life years (DALYs) due to nervous system disorders for adults aged ≥20 years [1,2]. Ischemic stroke accounts for 87% of all strokes, and its global prevalence is around 70 million cases per year [2]. The approval of intravenous pharmacological thrombolysis in the mid-1990s, the increasing availability of endovascular thrombectomy, and the growth of comprehensive stroke units, especially in high-income countries, have probably contributed to the decreased DALYs associated with ischemic stroke [2]. Nevertheless, less than 10% of patients can actually benefit from pharmacological or mechanical reperfusion [1,3,4], underscoring the urgency of developing novel therapies aimed at rescuing brain tissue. An intriguing opportunity would consist in mimicking the processes involved in endogenous ischemic tolerance, a condition that develops as a consequence of a sub-lethal ischemic episode, as demonstrated both in patients who have undergone a transient ischemic attack [5,6,7,8,9,10] and in animal models [11,12]. In fact, ischemic preconditioning (PC) in rodents activates a complex cascade of events that prompt brain resilience to an ensuing severe ischemia [13,14,15]. Ischemic tolerance involves the preservation of ionic homeostasis and mitochondrial function, the suppression of excitotoxic and inflammatory reactions, and interference with cell demise and blood–brain barrier damage, fostering brain recovery and regeneration [16,17,18,19,20,21,22].

Although the adaptive responses triggered by ischemic PC were originally believed to predominantly occur in neurons [23,24,25], more recent work has originally demonstrated the crucial involvement of the immune system in the establishment of the tolerant phenotype, in both preclinical and clinical settings [26]. In particular, Toll-like receptors (TLRs)- and Type-I interferon (INF)-mediated pathways, the release of inflammatory cytokines, as well as the genomic reprogramming of microglia towards reparative and protective M2-like phenotypes have been implicated in the protective effects of ischemic PC in rodents [27,28,29,30]. Indeed, despite their inflammatory function, microglia also exert a beneficial/reparative role in cerebral ischemia and/or trigger PC-induced tolerance via the stimulation of the fractalkine receptor CXCR1 [28,31,32,33,34]. Notably, we have recently observed that neuroprotection exerted by ischemic PC in mice is associated with a complex modulation of the innate immune system that results in the potentiation of M2-like responses in myeloid cells migrating from the periphery to the lesioned brain [35]. Thus, it is now clear that an intricate neuro-immune interplay underlies tolerance induced by PC [36,37,38].

In order to further delineate these mechanisms, we have focused on tumor necrosis factor (TNF)-α-stimulated gene 6 (TSG-6), a hyaluronate (HA)-binding protein involved in cell–cell and cell–matrix interactions during inflammation and tumorigenesis [39,40,41]. Interestingly, by blocking the pentraxin-3/fibroblast growth factor-2 interaction, TSG-6 was found to hamper vascular inflammation and endothelial dysfunction [42], two pivotal pathobiological mechanisms implicated in the progression of ischemic stroke and relevant co-morbidities (e.g., COVID-19), as well as in the development of long-term consequences (e.g., cognitive impairment) [43,44,45,46,47]. Most studies underscore the role of TSG-6 in the regulation of immune functions, which has recently been shown to underlie its anti-inflammatory effects in diverse neuropathological conditions [48,49,50]. Besides the evidence that TSG-6 promotes a microglial polarization shift from the M1 to M2 phenotype, there is also its ability to mediate the therapeutic efficacy of mesenchymal stem cells (MSCs) in various pathological contexts by exerting immunomodulatory effects [49,51]. Notably, the anti-inflammatory and immunosuppressive effects of TSG-6 were also demonstrated in acute neurological conditions, whereby the efficacy of MSCs was diminished by the siRNA knockdown of TSG-6 and/or treatment with recombinant TSG-6 exerted similar therapeutic activity [52,53,54]. In addition, in rodent models of focal cerebral ischemia, TSG-6 mediates the protective effects conferred by the systemic administration of MSCs [55], while systemic acute treatment with recombinant TSG-6 exerts neuroprotection [48]. Since neuroprotection induced by a sub-lethal ischemic insult involves the modulation of inflammatory and immune responses [35], we hypothesized that TSG-6 may represent a crucial immunoregulatory player underlying ischemic tolerance. Thus, using a murine model of transient middle cerebral artery occlusion (MCAo), we assess whether neuroprotection prompted by ischemic PC results in the modulation of TSG-6 in the brain and plasma.

## 2. Materials and Methods

### 2.1. Animals

Experiments were performed on adult C57Bl/6J male mice (Charles River, Calco, Como, Italy), weighing 25 +/− 3 g (9–11 weeks old). Animal housing occurred under standard conditions (22 °C ambient temperature, 65% relative humidity and 12 h light:12 h dark cycle), with ad libitum access to food and water.

The in vivo procedures were conducted respecting the guidelines of the Italian Ministry of Health (DL 26/2014), in conformity with the 2010/63 European Directive, and the experimental protocols (n. 975/2017-PR and 701/2020-PR) were approved by the local animal welfare organism (OBPA, University of Calabria) and by the Ministry of Health at the National Institute of Health (Rome). All animal procedures were performed in accordance with the NIH Guide for the Care and Use of Laboratory Animals and the Animal Research: Reporting of in vivo Experiments (ARRIVE) guidelines 2.0 [56].

An a priori power analysis was used to estimate the minimum sample size, aimed at obtaining a power of 80% at a significance level of 0.05 (OpenEpi software 3.01, Open Source Statistics for Public Health). Based on our previous experience with the focal ischemia model, we hypothesized a difference in cerebral lesion between mice who have undergone MCAo and animals receiving a neuroprotective treatment of about 25 mm^3^ (corresponding to a 25% reduction in infarct size) and a variability (standard deviation) of 15. This led to an estimated minimum sample size of 6 animals per experimental group. Considering a 30% attrition rate, corrected sample size was calculated as estimated sample size/(1 − [% attrition/100]). Therefore, 9 mice were assigned to each experimental group requiring statistical analysis, while 3 mice were assigned to immunofluorescence experiments finalized at qualitative analysis.

Sixty mice were randomly allocated to the following experimental groups:

(1) SHAM: SHAM surgery followed by sacrifice 24 h later (n = 9 for molecular biology analysis in plasma and brain);
(2) PC: 15 min MCAo followed by 72 h of reperfusion (n = 9 for molecular biology analysis in plasma and brain);
(3) MCAo: 1 h MCAo (preceded by SHAM surgery 72 h before) followed by 24 h of reperfusion (n = 9 for molecular biology analysis in plasma and brain + n = 9 for histology + n = 3 for immunofluorescence);
(4) PC + MCAo: 15 min MCAo followed, 72 h later, by 1 h MCAo and 24 h of reperfusion (n = 9 for molecular biology analysis in plasma and brain + n = 9 for histology + n = 3 for immunofluorescence).

### 2.2. Surgical Procedure for MCAo

Focal brain ischemia was induced by the proximal occlusion of the middle cerebral artery, using a well-established procedure [57,58,59] in mice anesthetized with 1.5–2% isoflurane vaporized in air. Briefly, a silicone-coated nylon filament (diameter: 0.23 mm, Doccol Corporation, Redlands, CA, USA) was advanced through the internal carotid artery for 10–11 mm from its bifurcation from the common carotid artery up to the origin of the middle cerebral artery in the Willis circle, proven by a mild resistance. Animals were considered ischemic, and included in the study, if presenting > 3 of the following deficits assessed after 45 min MCAo: ellipsoidal shape of the palpebral fissure, lateral extension of one or both ears, asymmetric body bending, laterally extending limbs [60]. Five animals were excluded from the study because of unsuccessful MCAo.

Ischemic PC was achieved through a previously demonstrated paradigm [11,20,28,61,62], by briefly (15 min) occluding the middle cerebral artery, 72 h before a more severe ischemia of 1 h MCAo (PC + MCAo). SHAM-operated control mice underwent the same surgical procedures without filament insertion. Four animals were excluded from the study because they died during or early after surgery.

To assess cerebral ischemic lesion, frozen brains were sectioned into 20 μm-thick coronal slices at 0.5 mm intervals from the frontal pole. Tissue slices were then stained with cresyl violet, and infarct (pale) areas were assessed by an investigator blinded to treatment allocation using an image analysis software (ImageJ, version 1.30), as previously described [63]. The percentage of infarction (infarct ratio) was calculated by dividing the infarcted tissue volume by the total volume of the ipsilateral hemisphere.

### 2.3. Western Blot Analysis

Animals were deeply anesthetized with isoflurane and sacrificed to dissect the ipsilateral (ischemic) and contralateral frontoparietal cortices (3.2 to −3.8 mm from Bregma) [64]. Blood was collected in EDTA Vacutainer^®^ tubes and centrifuged at 1500× *g* for 15 min at 4 °C to separate plasma, followed by centrifugation at 12,000× *g* for 15 min at 4 °C to discard debris and insoluble particles.

Tissue samples were homogenized in ice-cold RIPA buffer supplemented with a protease inhibitor cocktail (PI, Sigma-Aldrich, Milan, Italy). After the centrifugation (for 15 min at 20,817× *g* at 4 °C) of the lysates, supernatants were assayed for protein quantification (Bradford protein assay, Bio-Rad Laboratories, Segrate, Italy), and 30 μg of proteins were mixed in Laemmli buffer (Sigma-Aldrich). Plasma samples were diluted (1:10) in ice-cold RIPA buffer supplemented with PI and mixed (10 μL: 10 μL) in Laemmli buffer. Protein samples were then loaded onto Mini-PROTEAN® TGX™ Precast Protein Gels for separation in a Mini-PROTEAN Tetra Cell apparatus (Bio-Rad Laboratories). Gels were finally electroblotted using the Trans-Blot Turbo transfer apparatus and Nitrocellulose Transfer kit (Bio-Rad Laboratories). Nitrocellulose membranes were stained with red Ponceau S solution (Sigma-Aldrich), and the image of albumin band (66 KDa) was captured using the iBright™ FL1500 Imaging System (Thermo Fisher Scientific, Milan, Italy). Membranes were then pre-incubated in blocking buffer (5% non-fat milk in 0.05% Tween-20 TRIS-buffered saline) for 1 h at room temperature and then incubated overnight, at 4 °C, with the following primary antibodies: mouse anti-TSG-6 (1:1000; MABT108, Millipore, Milan, Italy) and mouse anti-β-actin (1:3000; A3853, Sigma-Aldrich), followed by the corresponding secondary antibodies (1:3000; Sigma-Aldrich) for 1 h at room temperature. The immunodetection of protein bands and quantification band density were performed using the iBright™ FL1500 Imaging System and ImageJ v1.51 software.

### 2.4. Real-Time Polymerase Chain Reaction (PCR)

MicroRNAs were extracted from plasma samples (collected as described above) using the miRNeasy Serum/Plasma Kit (Qiagen, Inc., Milan, Italy), as previously reported [35]. Briefly, samples were mixed with 5 volumes of QIAzol lysis reagent and 5 pM *A. Thaliana* miR-159a (478411_mir, Thermo Fisher) used as spike-in control. The solution was mixed with chloroform and centrifuged (12,000× *g* for 15 min at 4 °C) to obtain and isolate the colorless upper aqueous phase. The latter was mixed with 1.5 volumes of 100% ethanol and dispensed into an RNeasy MinElute spin column, to then be centrifuged at 8000× *g* for 15 s at room temperature. After washing the column with buffers (RWT and RPE) and 80% ethanol, miRNAs were eluted in 14 μL RNase-free water.

Finally, miRNAs were quantified using the TaqMan Advanced miRNA Assays Kit (Thermo Fisher Scientific) on a QuantStudio™ 3 real-time PCR system (Thermo Fisher Scientific). By using the comparative cycle threshold (Ct) method, the relative expression levels of *miR-23a-3p* (mmu478532_mir, Thermo Fisher), *miR-23b-3p* (mmu478602_mir, Thermo Fisher), and *miR-744-3p* (mmu482884_mir, Thermo Fisher) were calculated by normalization to the expression of *miR-669c-3p* (mmu483332_mir, Thermo Fisher) that was stable in all of the tested samples.

### 2.5. Immunofluorescence

Paraformaldehyde-fixed brains were sectioned into 40 µm-thick coronal slices obtained from the areas perfused by the middle cerebral artery (1.18 to −0.10 mm from Bregma) [64]. As previously reported [65,66], tissue sections were incubated with the following primary antibodies: rabbit polyclonal anti-TNFAIP6 (1:200 dilution; PA599494, Thermo Fisher), rat monoclonal anti-Ly-6B.2 (1:200 dilution; clone 7/4; AbD Serotec, Segrate, MI, Italy) to label myeloid cells (i.e., granulocytes and monocytes/macrophages); rat monoclonal anti-GFAP (1:150 dilution; clone 2.2B10, Invitrogen, Milan, Italy) to label astrocytes. Primary antibodies were labeled with AlexaFluor 488- or AlexaFluor 568-conjugated secondary antibodies (1:200 dilution; Invitrogen, Thermo Fisher Scientific, Milan, Italy); 4′,6-diamidino-2-phenylindole (DAPI, 1:500; Sigma-Aldrich, Milan, Italy) was used to counterstain nuclei. Fluorescence signals were acquired with a confocal laser scanning microscope (Fluoview FV300, Olympus, Segrate, MI, Italy).

### 2.6. Statistical Analysis

Data are expressed as mean ± S.E.M. and were subjected to statistical analysis using Graph-Pad Prism software for Windows (version 6.0, GraphPad Software, San Diego, CA, USA). Comparisons between two experimental groups were performed by unpaired Student’s *t* test, whereas comparisons between multiple experimental groups were performed using one- or two-way ANOVA followed by Tukey or Bonferroni post-tests, respectively. Values of *p* < 0.05 were considered to be significant.

## 3. Results

**Figure 1:**
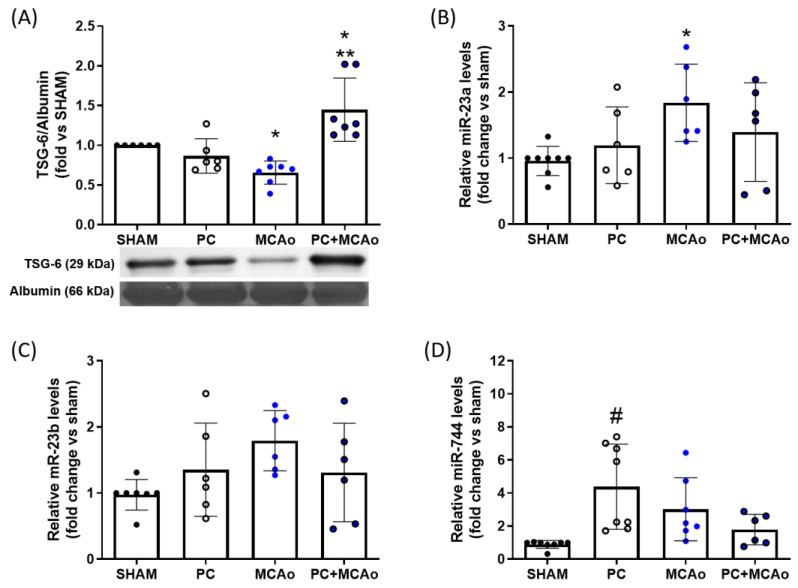
Transient MCAo and ischemic PC modulate plasma levels of TSG-6 protein and of miR-23a, miR-23b, and miR-744. (**A**) TSG-6 protein, (**B**) miR-23a, (**C**) miR-23b, or (**D**) miR-744 relative expression levels in plasma of mice who have undergone SHAM surgery (SHAM), PC (15 min MCAo), transient (1 h) MCAo, or PC + MCAo. * *p* < 0.05 vs. SHAM, ** *p* < 0.01 vs. PC and *p* < 0.001 vs. MCAo, # *p* < 0.001 vs. SHAM (one-way ANOVA followed by Tukey’s post-test, n = 6–8 mice per experimental group).

In order to assess whether ischemic PC affects circulating TSG-6, we have measured expression levels of this protein in the plasma of mice subjected to a severe ischemic insult (24 h MCAo followed by 24 h reperfusion), preceded or not by a preconditioning challenge (15 min MCAo) 72 h before. As shown in Figure 1A, transient 1 h MCAo resulted in a significant reduction in TSG-6 protein levels in plasma as compared with the SHAM condition. By contrast, pre-exposure to ischemic PC resulted in a significant elevation of TSG-6 plasma levels as compared with MCAo or PC alone (Figure 1A). We next assessed the modulation of plasma levels of miR-23a, miR-23b, and miR-744, identified by reverse target prediction analysis based on their potential to directly or indirectly regulate TSG-6 expression. By RT-PCR, we detected that ischemic PC abolished the elevation of miR-23a in the plasma mice exposed to transient 1 h MCAo (Figure 1B), which was coincident with reduced protein levels with respect to SHAM (Figure 1A). A similar trend was observed for miR-23b (Figure 1C) and miR-744 (Figure 1D), although the latter was notably elevated by the preconditioning stimulus alone, strongly suggesting its putative upstream role in TSG-6 regulation.

**Figure 2:**
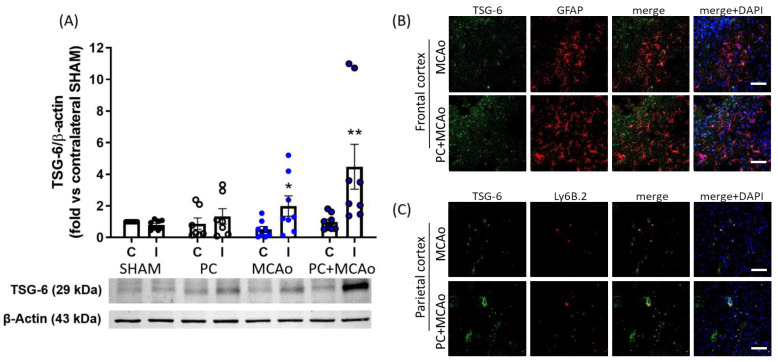
Ischemic PC potentiates elevation of TSG-6 protein expression in the brain of mice subjected to transient MCAo. (**A**) TSG-6 protein expression levels detected by Western blotting in the ipsilateral (ischemic, I) and contralateral (**C**) cortex of mice subjected to SHAM surgery (SHAM), PC (15 min MCAo), transient (1 h) MCAo, or PC + MCAo (** *p* < 0.01 vs. SHAM ipsilateral, vs. PC ipsilateral and vs. corresponding contralateral, * *p* < 0.05 vs. PC + MCAo ipsilateral (two-way ANOVA followed by Bonferroni post-test, n = 6–8 animals per experimental group). (**B**) Representative immunofluorescence images of the ipsilateral frontal cortex showing colocalization (yellow overlapping in merge panels) of TSG6 (green fluorescence) with GFAP-immunopositive neurons (red fluorescence). (**C**) Representative immunofluorescence images of the ipsilateral parietal cortex showing colocalization (yellow overlapping in merge panels) of TSG-6 (green fluorescence) with Ly6B.2-immunopositive myeloid cells (i.e., granulocytes and monocytes/macrophages, red fluorescence). Nuclei were counterstained with DAPI (blue signal). Scale bars = 150 μm.

**Figure 3:**
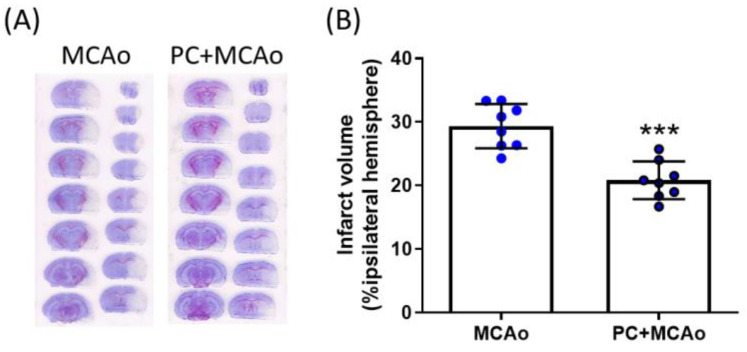
Ischemic PC reduces cerebral infarct volume caused by transient MCAo in mice. (**A**) Representative cresyl violet-stained coronal brain slices and (**B**) infarct damage expressed as percentage of ipsilateral hemisphere of mice subjected to 1 h MCAo followed by 24 h reperfusion, preceded (PC + MCAo) or not (MCAo) by 15 min MCAo, 72 h before. *** *p* < 0.0001 vs. MCAo (Student’s *t* test, n = 8 animals per experimental group).

In order to assess whether the peripheral modulation of TSG-6 was associated with a cerebral response, we next evaluated TSG-6 protein expression levels in brain cortical homogenates. Western blotting analysis revealed that 1 h MCAo resulted in a 1.99-fold elevation of TSG-6 protein levels in the ischemic cortex as compared to contralateral tissue (Figure 2A). This effect was further potentiated (2.25 fold vs. MCAo) in the ipsilateral cortex of mice pre-exposed to ischemic PC, strengthening the hypothesis that TSG-6 plays a role in ischemic tolerance. To characterize the specific cellular expression of this protein, we then performed immunofluorescence co-localization experiments in brain slices from mice subjected to MCAo preceded or not by ischemic PC. As shown in Figure 2B, TSG-6 was mainly detected in the nuclear and cytoplasmic compartments of GFAP-immunopositive astrocytes populating the ipsilateral frontal cortex (ischemic penumbra). By contrast, in the ipsilateral parietal cortex (ischemic core), the expression of TSG-6 was observed in Ly6B.2-immunopositive myeloid cells (Figure 2C). Given the immunoregulatory role of TSG-6, and its ability to mediate the beneficial functions of astrocytes and myeloid cells in other pathological contexts [49,67,68], we have hypothesized that its elevation in these cell types may represent a crucial mechanism implicated in neuroprotection. Thus, we assessed brain infarct volume by staining coronal brain sections with cresyl violet and observed that ischemic PC reduced lesioned (unstained, pale) areas, especially in the penumbra regions (i.e., motor cortex and medial striatum) (Figure 3A). The quantification of infarct brain damage revealed that MCAo produced a lesion that occupies 28.5% of the ipsilateral hemisphere, while the damaged region in mice pre-exposed to ischemic PC was 20.4% of total hemisphere, corresponding to a significant 29% reduction in cerebral infarct volume provided by ischemic PC (Figure 3).

## 4. Discussion

The present study originally demonstrates that the immunoregulatory protein TSG-6 is involved in the establishment of tolerance triggered by ischemic PC. In particular, we have observed that the reduction in TSG-6 protein levels occurring in the plasma of mice subjected to 1 h MCAo is abolished by pre-exposure to a PC stimulus, with the latter even resulting in a dramatic elevation of the circulating protein as compared to the SHAM condition. The peripheral response coincided with a significant elevation of cerebral levels of TSG-6 detected in both astrocytes and myeloid cells populating the ipsilateral hemisphere of mice subjected to ischemia. The cerebral and peripheral modulation of TSG-6 by ischemic PC was associated with a significant reduction in cerebral infarct volume, strongly suggesting that this immunomodulatory protein takes part in the protective mechanisms implicated in ischemic tolerance. These results further support the beneficial role of TSG-6 in acute ischemic stroke.

Recent work has underscored the crucial role of TSG-6 in acute neurodegenerative diseases, including cerebral ischemia both in human and in preclinical setting [48,49,50,69,70]. TSG-6 is a hyaluronate (HA)-binding protein constitutively expressed in the brain and upregulated under inflammatory conditions in a multitude of cells, such as astrocytes, monocytes/macrophages, dendritic cells, fibroblasts, mesenchymal stem/stromal cells (MSCs), and vascular smooth muscle cells [68,71,72,73,74].

TSG-6 plays a role in the modulation of stromal cell function and regulates immune responses by interacting with cell surface receptors and soluble mediators (e.g., chemokines) [51], thus playing anti-inflammatory and immunosuppressive roles in various diseases, including neuroinflammation [75,76,77,78,79,80,81,82]. Accordingly, this protein protects tissues from the injurious effects caused by spinal cord injury [83] and acute cerebral injury [52,53,54,84,85,86]. Notably, in rodent models of focal and global brain ischemia, TSG-6 underlies the neuroprotective effects of systemically administered MSCs [55,86,87].

In the brain of stroke patients, the expression of TSG-6 is elevated in the peri-infarct and infarcted tissue areas [69], whereas, in non-cardioembolic acute ischemic stroke patients, the increase in plasma levels of TSG-6 was positively associated with disease severity and lesion volume [70]. Moreover, we have recently demonstrated that TSG-6 protein levels increase in the brain of mice subjected to transient 1 h MCAo from 6 to 48h after reperfusion [48]. Here, we confirm and extend this evidence by demonstrating that TSG-6 expression is further potentiated in the ipsilateral cortex of mice pre-exposed to ischemic PC, being mainly detected in astrocytes and myeloid cells populating the prefrontal and parietal cortex, namely penumbra and core regions, respectively. The expression of TSG-6 in astrocytes has previously been demonstrated both in the brain and in the spinal cord of rats, whereby it contributes to their activation after injury, being major constituents of the glial scar [68,88]. In the ischemic brain, glial scar formation by astrocytes provides a physical barrier, hampering synaptic regeneration and neuronal repair at very late stages. By contrast, during the acute phase, it plays a protective role by restricting the spread of inflammation [89]. This is consistent with the evidence that *TSG-6 null* mice display increased glial scar deposition and a more severe inflammatory response after traumatic brain injury when compared to littermate control mice [67]. Thus, in agreement with these previous findings, our observation of an elevated expression of TSG-6 in penumbra astrocytes of the lesioned hemisphere of mice pre-exposed to ischemic PC may actually suggest a beneficial role. In particular, the formation of a specialized HA-TSG-6 matrix has been argued to serve to stabilize the glial scar, establishing an immunosuppressive milieu that protects adjacent tissue from further damage [67,68]. In addition to astrocytes, the expression of TSG-6 was also reported to occur in neurons, microglia, and blood-borne myeloid cells, especially under neurodegenerative/inflammatory conditions [48,51,69,85,90]. Here, we observe that TSG-6 is expressed in Ly6B.2-immunopositive myeloid cells (i.e., granulocytes and monocytes/macrophages) populating the ipsilateral parietal cortex. As previously suggested, the elevation of Ly6B.2-/TSG-6-immunopositive cells may actually provide protective effects by promoting the phenotype switching of myeloid cells towards the reparative M2-like phenotype [49]. Accordingly, in models of focal cerebral ischemia–reperfusion injury, the administration of recombinant TSG-6 exerts neuroprotection by promoting Ym1 brain expression while suppressing endoplasmic reticulum stress-related inflammation [48,50]. Thus, we speculate that the elevated levels of TSG-6 triggered by ischemic PC in mice subjected to MCAo may actually underlie the anti-inflammatory and immunomodulatory effects of this neuroprotective paradigm in ischemic stroke [35].

The effect of ischemic PC on TSG-6 did not exclusively occur in the brain, since we observed that protein levels were also modulated in plasma. In particular, after 24 h of reperfusion, despite a reduction in plasma TSG-6 protein levels caused by 1 h MCAo, pre-exposure to the PC stimulus dramatically elevated its expression as compared to SHAM-operated mice. Thus, it could be speculated that the cerebral elevation of TSG-6 observed in the preconditioned brain may depend not only on its local release by astrocytes, but also by blood-borne infiltrating myeloid cells, likely representing a compensatory anti-inflammatory mechanism, as also underscored in other inflammatory contexts [91]. Conversely, we speculate that the reduced levels of circulating TSG-6 observed after transient MCAo may represent a trigger for a detrimental response to injury. To support this hypothesis, we have previously demonstrated that the administration of recombinant TSG-6 at the time of reperfusion caused the significant elevation of cerebral levels of the M2 marker Ym1 and a reduction in cerebral lesion and neurological deficits in mice subjected to transient MCAo [48]. In turn, the knockdown of endogenous TSG-6 by siRNA elevated the (CD86+) M1 vs. (CD163+) M2 ratio in cerebral microglia and aggravated neurological deficits 24 h after subarachnoid hemorrhage [85,92]. In fact, inflammatory stimuli prompt myeloid immune cells to produce high levels of TSG-6 [93], that, in turn, can act in an autocrine mode on macrophages triggering their transition from inflammatory to anti-inflammatory and immunoregulatory phenotypes [94,95,96,97], likely through the suppression of Toll-like receptor (TLR)-4/NF-κB pathways [58,76,78,95,98,99]. Thus, the anti-inflammatory and immunomodulatory functions of TSG-6 may be crucial for the neuroprotective effects of ischemic preconditioning. In this regard, it is important to highlight that preconditioning alone is not sufficient to modulate cerebral and peripheral TSG-6 levels, suggesting the occurrence of an upstream mechanism that combines with severe ischemia to then produce the protective effect. Using the TargetScan database (www.targetscan.org, accessed on 14 September 2021), we performed a reverse target prediction analysis to identify miR-23a and miR-23b based on their potential to regulate TSG-6. As expected, the elevation of miR-23a expression was coincident with reduced TSG-6 protein expression after 1 h MCAo followed by 24 h reperfusion. By contrast, in mice pre-exposed to ischemic PC, the elevation of TSG-6 did not correspond to a significant modulation of miR-23a, suggesting that protein increase may actually be independent from miRNA regulation. Moreover, based on previous findings, we have screened other miRNAs potentially involved in the modulation of TSG-6 to finally identify miR-744 that can induce TSG-6 expression via the inhibition of TGF-beta synthesis [100,101,102,103,104]. Interestingly, the miR-744 expression level was significantly elevated after the PC stimulus alone, arguing for its crucial role in ischemic tolerance. Accordingly, the systemic administration of miR-744 to rats subjected to focal cerebral ischemia was able to provide neuroprotection by suppressing inflammatory responses (i.e., elevation of TNF-α, IL-1-β and IL-6) in the injured brain [105]. The same authors demonstrated that cerebral miR-744 levels are decreased after ischemia–reperfusion as compared to SHAM, further supporting the beneficial role of this miRNA and its downstream pathways [105].

## 5. Conclusions

Although our findings provide information on TSG-6 protein levels, further work is needed to elucidate in which (specifically sorted) immune cells the modulation of its mRNA occurs. Moreover, our study only provides preliminary findings on the putative role of TSG-6 in ischemic tolerance in adult male mice, since the lack of investigation of sex differences (i.e., by including female mice), aging, co-morbidities, or long-term parameters represent important limitations.

In conclusion, our study demonstrates that ischemic PC protects against a more severe ischemia by modulating cerebral and plasma levels of TSG-6, an immunomodulatory protein likely involved in ischemic tolerance. This further strengthens the crucial role of TSG-6 in the ischemic context, highlighting its potential as a promising therapy for neuroprotection in stroke.

## Data Availability

Data are contained within the article.
